# Isolation and Identification of Allelopathic Substances from *Forsythia suspensa* Leaves, and Their Metabolism and Activity

**DOI:** 10.3390/plants13050575

**Published:** 2024-02-20

**Authors:** Hisashi Kato-Noguchi, Yuga Takahashi, Shunya Tojo, Toshiaki Teruya

**Affiliations:** 1 Department of Applied Biological Science, Faculty of Agriculture, Kagawa University, Miki 761-0795, Kagawa, Japan; s20a068@kagawa-u.ac.jp; 2 Graduate School of Engineering and Science, University of the Ryukyus, 1 Senbaru, Nishihara 903-0213, Okinawa, Japan; k23803@eve.u-ryukyu.ac.jp; 3 Faculty of Education, University of the Ryukyus, 1 Senbaru, Nishihara 903-0213, Okinawa, Japan; t-teruya@edu.u-ryukyu.ac.jp

**Keywords:** arctigenin, eco-friendly agriculture, lignan, matairesinol, soil additive, pinolesinol, pruned waste

## Abstract

The fruit of *Forsythia suspensa* (Thunb.) Vahl has been used in traditional Chinese medicine as “*Forsythiae fructus*”. The species is also grown in parks and gardens, and on streets and building lots, as an ornamental plant, but it requires pruning. In this study, the allelopathic activity and allelopathic substances in the leaves of pruned branches of *F. suspensa* were investigated to determine any potential application. The leaf extracts of *F. suspensa* showed growth inhibitory activity against three weed species; *Echinochloa crus-galli*, *Lolium multiflorum,* and *Vulpia myuros.* Two allelopathic substances in the extracts were isolated through the bioassay-guided purification process, and identified as (-)-matairesinol and (-)-arctigenin. (-)-Matairesinol and (-)-arctigenin, which showed significant growth inhibitory activity at concentrations greater than 0.3 mM in vitro. The inhibitory activity of (-)-arctigenin was greater than that of (-)-matairesinol. However, both compounds were more active than (+)-pinolesinol which is their precursor in the biosynthetic pathway. The investigation suggests that *F. suspensa* leaves are allelopathic, and (-)-matairesinol and (-)-arctigenin may contribute to the growth inhibitory activities. Therefore, the leaves of the pruned branches can be applied as a weed management strategy in some agricultural practices such as using the leaf extracts in a foliar spray and the leaves in a soil mixture, thereby reducing the dependency on synthetic herbicides in the crop cultivation and contributing to developing eco-friendly agriculture.

## 1. Introduction

*Forsythia suspensa* (Thunb.) Vahl belongs to the Oleaceae family. The genus of *Forsythia* consists of about 10 species, which are native to eastern Asia except one species (*Forsythia europaea)* that is native to southern Europe. *F. suspensa* is native to China, is a deciduous shrub with 1 m–3 m high. The opposite leaves are usually simple, but occasionally three-foliolate. Leaf blades are ovate-oblong, 6 cm–10 cm long and 1.5 cm–5 cm wide with a rounded to cuneate base and an acute apex. The petioles are 0.8 cm–1.5 cm long. The species bears flowers at leaf axils with a 5 mm–6 mm long pedicel. The corollas are yellow and divided into four lobes, and the lobes are 1.2 cm–2 cm long and 0.6 cm–1 cm wide. Pistils are 5 mm–7 mm long with 3 mm–5 mm long stamens. The fruits (capsules) are ovoid to ellipsoid, 1.2 cm–2.5 cm long and 0.6 cm–1.2 cm wide with 0.7 cm–1.5 cm long stalks [1,2,3]. Its chromosome number is 2n = 48 [4,5]. The species grows in grassy and thicket areas in valleys and on slopes at 300 m–2200 m above sea level in the warm temperature zones [1,6]. Some of the *F. suspensa* population showed cold- and/or drought tolerance [6].

The dried fruits of the species have been used in traditional Chinese medicine as “*Forsythiae fructus*”. *Forsythiae fructus* has a bitter flavor, and has been traditionally applied for thousands of years as an effective remedy for the treatments of carbuncle, scrofula, gall tumor, malignant ulcer, gonorrhea, nephritis, and erysipelas. This medicinal herb is effective in clearing heat channel and releasing the heart of stomach and spleen according to traditional medicine [1,7,8]. Recent investigations have shown that *Forsythiae fructus* has several pharmacological activities such as anti-inflammatory, antibacterial, anti-allergic, anti-cancer, anti-HIV, antioxidant, and neuroprotective activities in vivo and in vitro [1,7,8,9]. More than 300 compounds in many chemical classes such as lignans, phenylethanoid glycosides, flavonoids, terpenoids, steroids and alkaloids have been identified in *Forsythiae fructus*, and the pharmacological activity of some of these compounds has been demonstrated [1,7,9].

**Figure 1:**
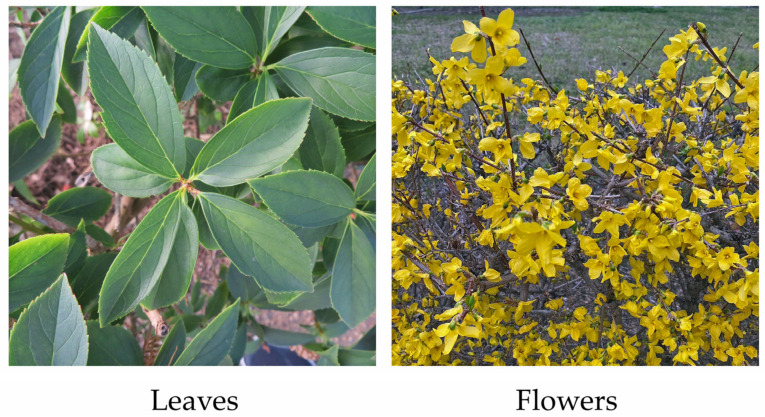
Leaves and flowers of *F. suspensa*.

**Figure 2:**
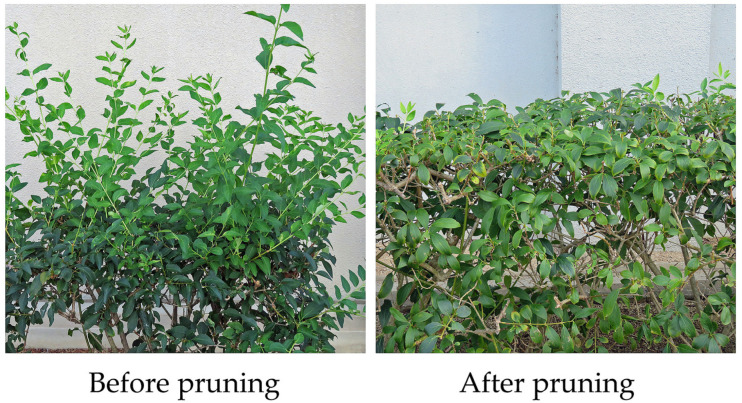
Before and after the pruning of *F. suspensa*.

*F. suspensa* is also cultivated widely in East Asia, Europe and North America in parks and gardens, and on streets and building lots as an ornamental plant because of its beautiful flowers [10,11,12,13]. The species blooms in early spring before the leaves appear, and the whole tree looks to be golden, which is attractive to people as an ornamental plant species (Figure 1). However, the plant grows vigorously and its branches straggle pendulously. Therefore, pruning is necessary to maintain the species as an ornamental plant (Figure 2) [10,11,12,13]. Pruning produces a large amount of waste because the plant is fast-growing. Consequently, developing a beneficial application of the pruned waste to help reduce the environmental and economic concerns is necessary. 

Many medicinal plants have been shown to possess relatively high allelopathic activity [14,15]. Allelopathy is the chemical interaction between donor plants and receiver plants through certain plant secondary metabolites defined as allelochemicals [16,17,18,19]. The allelochemicals can suppress the germination and growth of other plants including weed plant species [20,21]. Several allelopathic plant species are effective in weed management practices in agricultural fields [22,23,24,25]. Therefore, allelopathic plants can be useful as a weed management option to reduce the application of commercial herbicides. *F. suspensa* may also contain allelochemicals because it is used as a medicinal plant and contains many secondary metabolites, so its pruned waste may be effective in weed management practices. However, no information on the allelopathy and allelochemicals of *F. suspensa* is available. The objective of this research was to evaluate the allelopathic activity of *F. suspensa* leaves against three weed species; *Echinochloa crus-galli* (L.) P.Beauv., *Lolium multiflorum* Lam., and *Vulpia myuros* (L.) C.C.Gmel., and to isolate and characterize the allelochemicals in the leaves of the pruned branches to develop a beneficial application of the pruned waste.

## 2. Results

### 2.1. Allelopathic Activity of the F. suspensa Leaves

**Figure 3:**
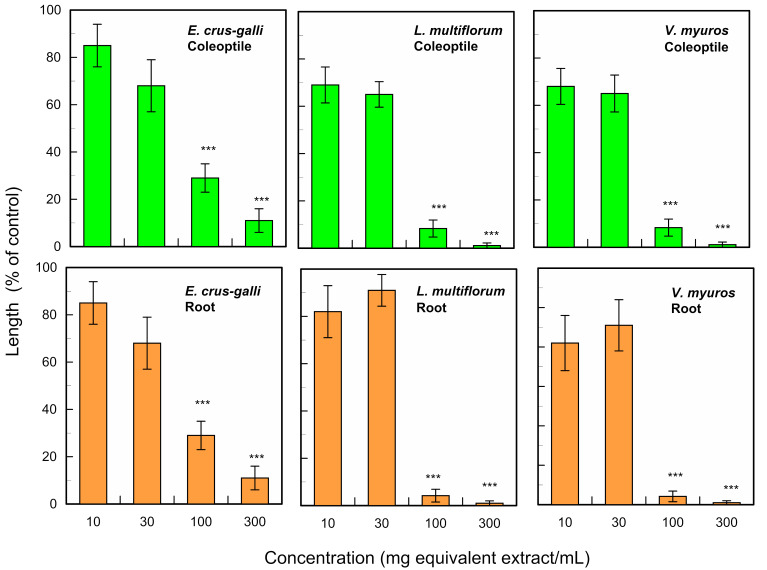
Effects of the leaf extracts of *F. suspensa* on the growth of the roots and coleoptiles of *E. crus-galli*, *L. multiflorum* and *V. myuros*. Concentration (mg leaf equivalent extract/mL) corresponds to the amount of the extracts obtained from 10, 30, 100 or 300 mg leaves per mL. Mean ± SE from 4 independent experiments with 10 seedlings for each determination are shown. Asterisks indicate significant differences between control and treatment: ***, *p* < 0.001.

**Figure 4:**
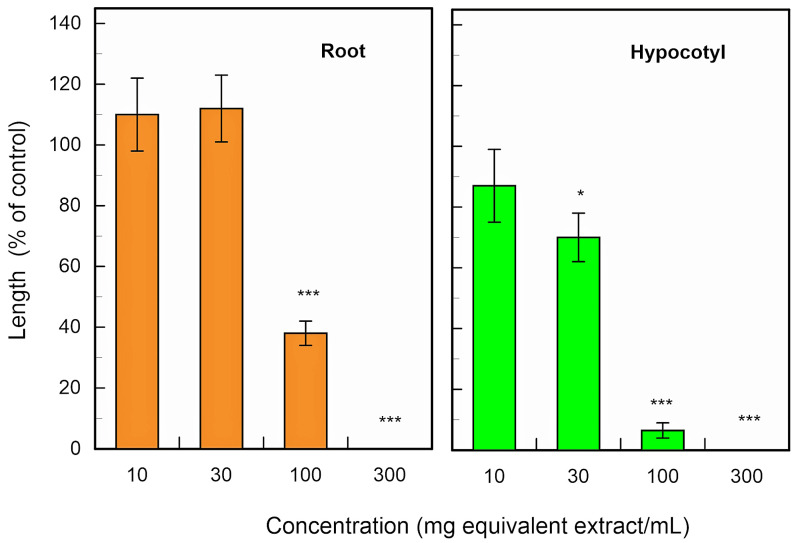
Effects of the *F. suspensa* leaf extracts on the growth of the roots and coleoptiles of cress. Other conditions were as described in Figure 3. Asterisks indicate significant differences between control and treatment: *, *p* < 0.05, ***, *p* < 0.001.

The leaf extracts of *F. suspensa* significantly inhibited the growth of the roots and coleoptiles of *E. crus-galli*, *L. multiflorum* and *V. myuros* at concentrations higher than 100 mg leaf extract per mL (Figure 3). The concentrations of the extracts causing 50% growth inhibition (*IC*_50_ values) of the extracts for the root growth were 3.8 mg, 39.4 mg and 12.4 mg leaf equivalent extract per mL for *E. crus-galli*, *L. multiflorum* and *V. myuros*, respectively, and the *IC*_50_ values for the coleoptiles were 43.9 mg, 46.1 mg and 18.1 mg leaf extract per mL for *E. crus-galli*, *L. multiflorum* and *V. myuros,* respectively. The extracts also inhibited the roots and hypocotyls of cress (*Lepidum sativum* L.) at concentrations greater than 100 mg and 30 mg leaf extract per mL for roots and hypocotyls, respectively (Figure 4). The *IC*_50_ values of the extracts against the cress roots and hypocotyls were 91.5 mg and 40.1 mg leaf equivalent extract per mL, respectively.

### 2.2. Purification and Identification of the Allelochemicals in the Leaves

**Figure 5:**
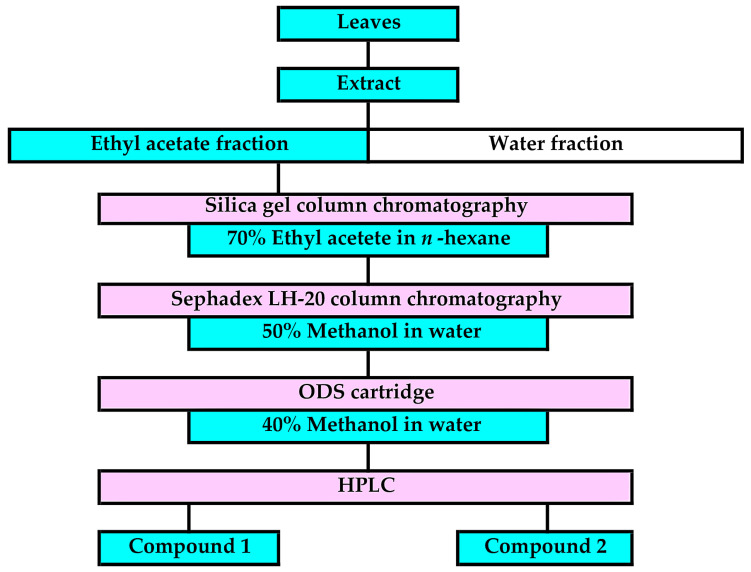
Purification process of compounds 1 and 2 from the leaf extract of *F. suspensa*.

**Figure 6:**
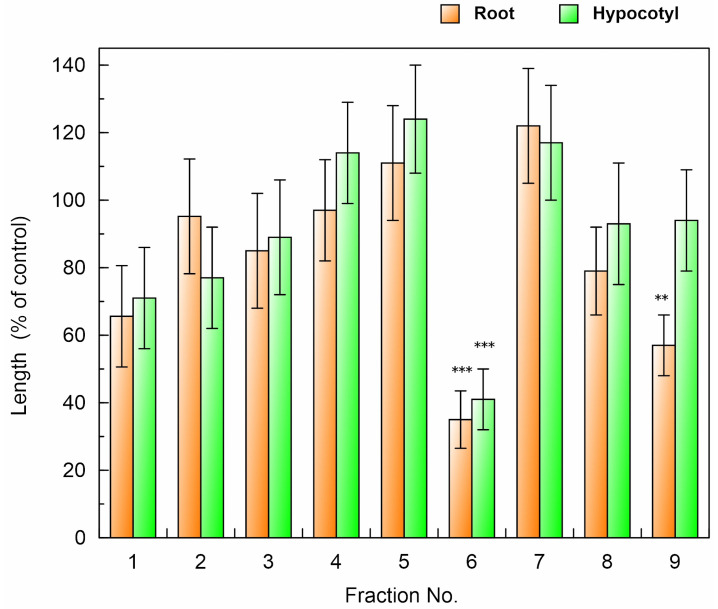
Effects of the fractions separated by a silica gel column chromatography on the root and hypocotyl growth of cress. Concentration of tested samples corresponded to the extract obtained from 0.3 g of *F. suspensa* per mL. Other conditions were as described in Figure 3. Asterisks indicate significant differences between control and treatment: **, *p* < 0.01, ***, *p* < 0.001.

**Figure 7:**
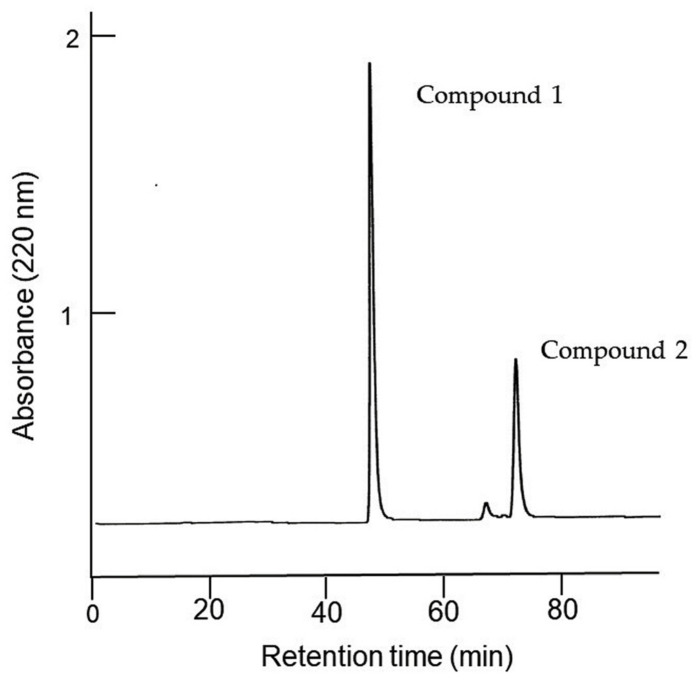
HPLC chromatogram and chemical structures of compounds 1 and 2. HPLC condition; column: reverse-phase, Inertsil ODS-3, 4.6 mm i.d. × 250 mm in length (YMC Ltd., Kyoto, Japan), solvent: 70% aqueous methanol (flow rate, 0.8 mL), detection: 220 nm.

The extract of the *F. suspensa* leaves was purified through the bioassay-guided purification process outlined in Figure 5. The ethyl acetate fraction was separated through silica gel chromatography, and the activity of all the separated fractions was determined using a cress bioassay. Allelopathic activity was found in fractions 5 and 9 (Figure 6). However, the fraction 9 significantly inhibited the growth of cress roots but not the hypocotyls. Fraction 5 inhibited the growth of the cress roots and hypocotyls to 35% and 41% of the control roots and hypocotyls, respectively. Therefore, the fraction 5 was further purified using a Sephadex LH-20 and ODS cartridge (Figure 5), and two compounds, 1 and 2, showing allelopathic activity were isolated using HPLC (Figure 7). 

**Figure 8:**
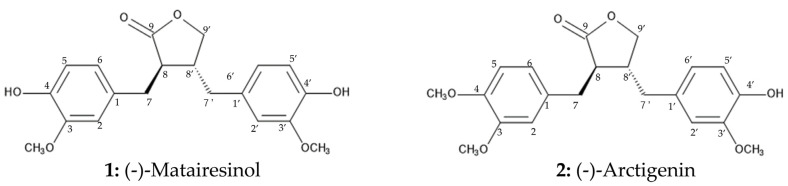
Chemical structures of (-)-matairesinol and (-)-arctigenin.

The molecular formula of compound 1 was determined as C_20_H_22_O_6_ by HRESI-MS. The ^1^H NMR spectrum showed the presence of six aromatic proton signals at *δ*_H_ 6.81 (1H, d, *J* = 7.9), 6.80 (1H, d, *J* = 8.0), 6.61 (1H, d, *J* = 1.7), 6.60 (1H, dd, *J* = 7.9, 1.7), 6.51 (1H, dd, *J* = 8.0, 1.7) and 6.41 (1H, d, *J* = 1.7), two methyl proton signals at *δ*_H_ 3.82 (3H, s) and 3.81 (3H, s); and two methine proton and six methylene proton signals at *δ*_H_ 4.15 (1H, dd, *J* = 9.1, 7.4), 3.89 (1H, dd, *J* = 9.1, 7.3), 2.95 (1H, dd, *J* = 14.1, 5.3), 2.88 (1H, dd, *J* = 14.1, 7.0), 2.61 (1H, dd, *J* = 13.6, 6.7) and 2.58−2.42 (3H, m). The optical rotation was [*α*]^27^_D_ = −38.6 (*c* 0.07, acetone). Based on the spectrum data and published data in the literature [26,27], the chemical structure of compound 1 was characterized as (-)-matairesinol (Figure 8). 

The molecular formula of compound 2 was found to be C_21_H_24_O_6_ by HRESI-MS. The ^1^H NMR spectrum showed the presence of six aromatic proton signals at *δ*_H_ 6.82 (1H, d, *J* = 8.0), 6.75 (1H, d, *J* = 8.2), 6.64 (1H, d, *J* = 1.2), 6.61 (1H, dd, *J* = 8.0, 1.2), 6.55 (1H, dd, *J* = 8.2, 1.4) and 6.46 (1H, d, *J* = 1.4), three methyl proton signals at *δ*_H_ 3.85 (3H, s), 3.82 (3H, s) and 3.81 (3H, s); and two methine proton and six methylene proton signals at *δ*_H_ 4.14 (1H, dd, *J* = 8.9, 7.5), 3.89 (1H, dd, *J* = 8.9, 7.8), 2.95 (1H, dd, *J* = 14.1, 5.3), 2.90 (1H, dd, *J* = 14.1, 6.7), 2.64 (1H, dd, *J* = 13.4, 6.1) and 2.59−2.44 (3H, m). The optical rotation was [*α*]^27^_D_ = −20.6 (*c* 0.13, MeOH). Compound 2 was identified as (-)-arctigenin (Figure 8) through these spectrum data and published data in the literature [27].

### 2.3. Allelopathic Activity of the Isolated Compounds and Pinolesinol

**Figure 9:**
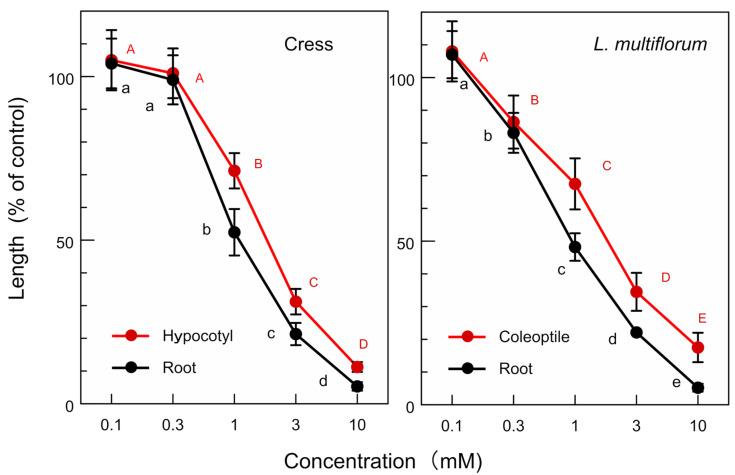
Effects of (-)-matairesinol on the growth of the roots and hypocotyls/coleoptiles of cress and *L. multiflorum.* Means ± SE from 4 independent experiments with 10 seedlings for each determination are shown. Different letters on the symbols in the same panels indicate significant differences at the *p* < 0.05 level.

**Figure 10:**
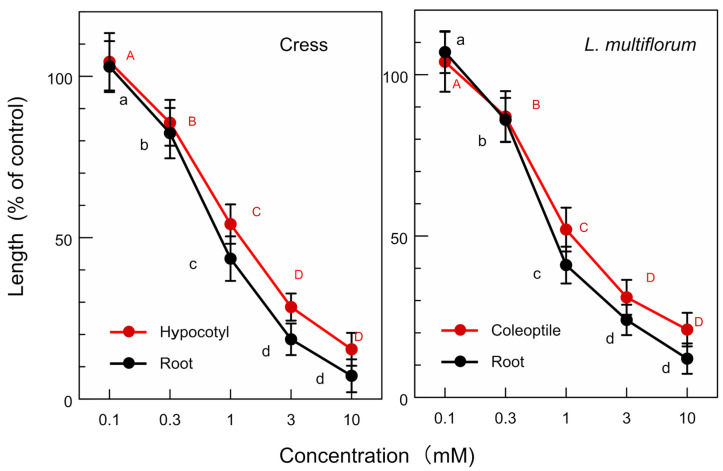
Effects of (-)-arctigenin on the growth of the roots and hypocotyls/coleoptiles of cress and *L. multiflorum.* Other conditions were as described in Figure 9.

**Figure 11:**
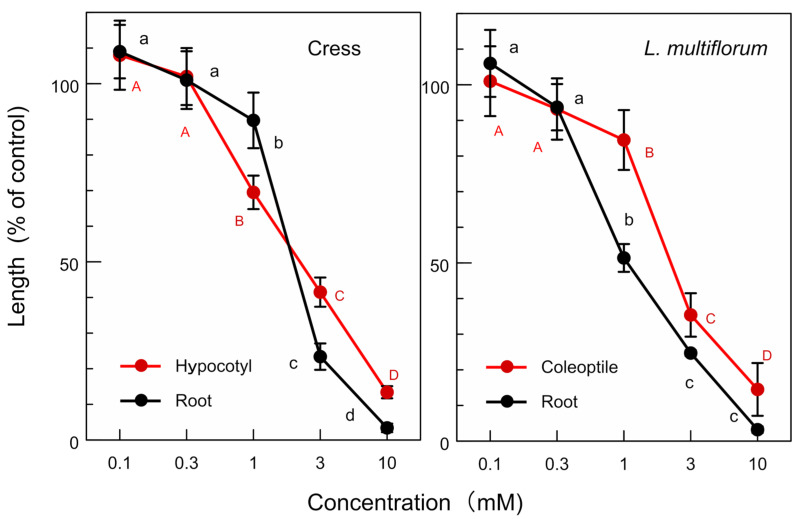
Effects of (+)-pinoresinol on the growth of the roots and hypocotyls/coleoptiles against of cress and *L. multiflorum.* Other conditions were as described in Figure 9.

The allelopathic activity of the isolated compounds, (-)-matairesinol and (-)-arctigenin, and their biosynthetic precursor, (+)-pinolesinol was determined. (-)-Matairesinol significantly inhibited the growth of the cress roots and hypocotyls at concentrations greater than 1 mM, and the growth of the *L. multiflorum* roots and coleoptiles at concentrations greater than 0.3 mM (Figure 9). The *IC*_50_ values of (-)-matairesinol against the cress roots and hypocotyls were 1.1 mM and 2.1 mM, respectively, and those for the *L. multiflorum* roots and coleoptiles were 0.93 mM and 2.2 mM, respectively (Table 1). (-)-Arctigenin also showed the growth inhibitory activity against the cress roots and hypocotyls, and the *L. multiflorum* roots and coleoptiles at concentrations greater than 0.3 mM (Figure 10). The *IC*_50_ values of (-)-arctigenin against the cress roots and hypocotyls were 0.79 mM and 1.3 mM, respectively, and those for the *L. multiflorum* roots and coleoptiles were 0.85 mM and 1.3 mM, respectively (Table 1). (+)-Pinolesinol was active at concentrations greater than 1 mM against the cress roots and hypocotyls, and *L. multiflorum* roots and coleoptiles (Figure 11). The *IC*_50_ values of (+)-pinolesinol for the cress roots and hypocotyls were 2.1 mM and 2.3 mM, respectively, and those for the *L. multiflorum* roots and coleoptiles were 1.1 mM and 2.5 mM, respectively (Table 1).

## 3. Discussion

As an ornamental tree, *F. suspensa* needs adequate pruning treatments because it is fast-growing [11,12,13], and developing a beneficial application of the pruned waste to help alleviate environmental and economic concerns is necessary. Accordingly, we determined the allelopathic activity of the leaves of *F. suspensa*. The leaf extracts inhibited the growth of the roots and coleoptiles of three weed species (Figure 3): the *E. crus-galli* roots were most sensitive to the extracts, and *L. multiflorum* coleoptiles were the least sensitive of the three weed species. Based on the growth inhibitory activity of the leaf extracts of *F. suspensa*, the leaves may contain certain allelochemicals. Allelochemicals are synthesized and accumulated in some plant tissues including leaves, and released into the neighboring environment including the rhizosphere soil through volatilization, root exudation, and decomposition processes of the donor plant residues in the soil [20,21,22,23]. Deciduous tree plants can also release allelochemicals into their rhizosphere soil over many years through the decomposition process of their fallen leaves [28,29,30]. *F. suspensa* is a deciduous plants species [1,2,3]. The compounds liberated from the fallen leaves may accumulate in the soil, and inhibit the germination and growth of other nearby plant species [31,32,33,34]. Therefore, allelochemicals in *F. suspensa* leaves may also be released into the neighboring environments and/or the rhizosphere soil.

The *F. suspensa* leaf extract was then purified through the bioassay-guided purification process (Figure 5), and two active compounds 1 and 2 were isolated (Figure 7). During the purification process, the allelopathic activity of all fractions obtained after each chromatography step was evaluated using a cress bioassay, and the fraction showed the highest activity was subjected to the next chromatography process as described by [35]. The germination rate of *E. crus-galli*, *L. multiflorum,* and *V. myuros* was 20–40% at 48 h after sowing, and was not stable during the experiments, while the germination rate of cress was 90% at 48 h after the sowing and stable during the experiments. Therefore, selecting germinated seeds for the cress bioassay is not necessary because of its high and stable germination behavior. Comparing the *IC*_50_ values of the extracts of the *F. suspensa* leaves against cress with *IC*_50_ values of the extracts against the three weed species showed that the sensitivity of the cress roots was low. However, the sensitivity of the cress hypocotyls was not high and not low. Therefore, cress was chosen as a bioassay species because of the convenience, and the growth of cress was monitored during extract purification. 

**Figure 12:**
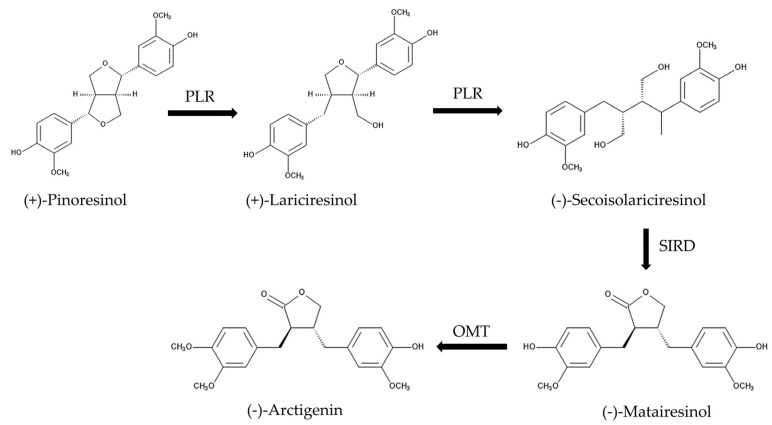
The biosynthetic pathway of (-)-matairesinol and (-)-arctigenin in the *Forsythia* species. PLR: pinoresinol/lariciresinol reductase, SIRD: secoisolariciresinol dehydrogenase, OMT: *O*-methyltransferases.

Compounds 1 and 2 were characterized as (-)-matairesinol and (-)-arctigenin, respectively, by the spectrum analysis of HRESI-MS, ^1^H-NMR and optical rotation (Figure 8). (-)-Matairesinol and (-)-arctigenin are classified as lignans. Lignans are a structurally diverse group of plant natural products originating from phenylpropanoids, have important physiological functions in plants, and exhibit pharmacological activity [36]. *Forsythia* species accumulate a considerable amount of lignans such as (+)-pinoresinol, (-)-matairesinol, (-)-arctigenin and their respective glycosides in the entire plant body [37]. The biosynthetic pathway of these lignans in *Forsythia species* has been reported (Figure 12). (+)-Pinoresinol is synthesized by radical coupling of two coniferyl alcohol molecules [38,39,40,41,42], and then converted to (-)-secoisolariciresinol via (+)-lariciresinol by pinoresinol/lariciresinol reductase (PLR).

(-)-Secoisolariciresinol is metabolized to (-)-matairesinol by secoisolariciresinol dehydrogenase (SIRD), and (-)-matairesinol is metabolized to (-)-arctigenin by *O*-methyltransferases (OMT). The majority of those lignans are glycosylated at their hydroxyl groups by a superfamily of enzymes; the uridine diphosphate (UDP)-sugar dependent glycosyltransferases (UGT). UGT transfers a sugar moiety from an activated UDP-sugar to an accepting substrate [37,38,39,40,41,42]. (-)-Matairesinol, (-)-arctigenin and (+)-pinoresinol have been reported to show several pharmacological activities such as anti-tumor, anti-HIV, anti-angiogenic, and estrogenic activity [36,43,44]. (+)-Pinoresinol isolated from Oleaceae species has also shown the growth inhibitory activity against several plant species as an allelopathic agent [35,45]. However, the allelopathic activity of (-)-matairesinol and (-)-arctigenin has not yet been reported.

Cress was selected as for a bioassay species for the isolated compounds because its growth could be monitored during extract purification. *L. multiflorum* was also selected as a bioassay species because its sensitivity to the *F. suspensa* extract was not high among the three weed species, *E. crus-galli*, *L. multiflorum* and *V. myuros* (Figure 3). (-)-Matairesinol and (-)-arctigenin suppressed the growth of the cress roots and hypocotyls and *L. multiflorum* roots and coleoptiles (Figure 9 and Figure 10). The active threshold of the growth inhibition of (-)-matairesinol was 1 mM and 0.3 mM for cress and *L. multiflorum,* respectively (Figure 9), and that of (-)-arctigenin was 0.3 mM for cress and *L. multiflorum* (Figure 10). The *IC*_50_ values of (-)-matairesinol and (-)-arctigenin against the growth of the hypocotyls/coleoptiles and roots of cress and *L. multiflorum* were 0.93 mM–2.2 mM and 0.79 mM–1.3 mM, respectively (Table 1). Therefore, the *IC*_50_ values and active threshold of the compounds show that the growth inhibitory activity of (-)-arctigenin was greater than that of (-)-matairesinol, which suggests that the methoxy group (CH_3_O-) at the C-4 position of (-)-arctigenin may increase the growth inhibitory activity compared with the hydroxy group (HO-) at the C-4 position of (-)-matairesinol (Figure 8). The *IC*_50_ values of their precursor in the biosynthetic pathway (Figure 12), (+)-pinolesinol were 1.1 mM–2.5 mM (Table 1). The active threshold of the growth inhibition of (+)-pinolesinol was 1 mM for cress and *L. multiflorum* (Figure 11). Comparing those *IC*_50_ values and the active threshold revealed that the activities of (-)-arctigenin and (-)-matairesinol were greater than (+)-pinolesinol. In addition, we isolated 26 mg of (-)-matairesinol and 35 mg of (-)-arctigenin from 100 g of *F. suspensa* leaves, which indicates the leaves may contain a relatively large amount of these compounds. Because (+)-lariciresinol and (-)-secoisolariciresinol (Figure 12) were not isolated from the extracts of the *F. suspensa* leaves through the bioassay-guided purification process (Figure 5), the growth inhibitory activity of both compounds may not be high compared with that of (-)-matairesinol and (-)-arctigenin. There have also been no reports on the plant growth inhibitory activity of these compounds. In addition, modifying the structure to increase allelopathic activity is possible [46].

When the leaf extracts and their soaking water obtained from some plant species such as *Lantana camara* L., *Imperata cylindrica* (L.) Beauv., *Solidago canadensis* L, *Tithonia diversifolia* (Hemsl.) A. Gray, *Bidens pilosa* L., and *Leucaena leucocephala* (Lam.) de Wit. were applied as foliar spray and/or irrigation water, the emergence and growth of several weed species were significantly inhibited under field, greenhouse and laboratory conditions [47,48,49,50,51,52,53,54,55]. When the leaves of *L. camara*, *I. cylindrica,* and *L. leucocephala* were mixed with soil, the emergence and growth of several weed species were also suppressed under field and greenhouse conditions [56,57,58,59,60]. These observations suggest that the leaf extracts and the soaking water of these plant species, and their residues incorporated into soil showed allelopathic activity against the emergence and growth of the weed species, and they may contain allelochemicals. Some of these allelochemicals in the residues may be liberated into the soil during the decomposition of the leaves [16,17,18,19,20,21,22,23]. Therefore, the leaves of allelopathic plants can be applied for the weed management purposes in eco-friendly agriculture. In addition, the allelochemicals of several plant extracts and residues also disturb this mutualism of weed plant species with rhizobia and/or arbuscular mycorrhizal fungi [31,36,60]. Rhizobium nodulation enhances the performance of legume plants through nitrogen supply [60,61]. Arbuscular mycorrhizal fungi enhance the performance of most territorial plants through increasing the absorption of nutrients, photosynthesis, and their defense functions [61,62,63,64,65]. The disturbance of this mutualism by allelochemicals may reduce the competitive ability of these weed species, and may relatively increase the competitive ability of crop plants. 

The present investigation suggests that the leaves of *F. suspensa* exhibit allelopathic activity against the growth of *E. crus-galli*, *L. multiflorum* and *V. myuros,* and contain the allelopathic substances, (-)-matairesinol and (-)-arctigenin. When the leaves of *F. suspensa* were incorporated into the soil as a soil mixture, certain allelochemicals in the leaves may be liberated into the field soil during the decomposition, and the liberated allelochemicals may suppress the emergence and growth of some weed species. The leaf extracts and its soaking water can be applied as a foliar spray to inhibit the emergence and growth of some weed plant species. The fruit of *F. suspensa* has been used in traditional Chinese medicine [1,7,8,9], and the leaves from the pruned branches of *F. suspensa* may also be useful for weed management.

## 4. Materials and Methods

### 4.1. Plant Material

*F. suspensa* leaves were obtained at the Kagawa University campus in April 2023. Three weed species; *Echinochloa crus-galli* (L.) P.Beauv., *Lolium multiflorum* Lam., and *Vulpia myuros* (L.) C.C.Gmel., were used to evaluate allelopathic activity. Cress (*Lepidum sativum* L.) was used during the purification process of the allelochemicals as a bioassay plant because of its convenient handling procedure and stable germination rate.

### 4.2. Extraction and Determination of the Allelopathic Activity of F. suspensa 

*F. suspensa* leaves (100 g fresh weight) were extracted by soaking in a mixture of water and methanol (20%: 80%, *v*/*v*, 1.5 L; Nacalai, Kyoto, Japan) for 48 h, and filtered using filter paper (No. 2; Advantec Toyo, Tokyo, Japan). The residue obtained from the first filtration was extracted again by soaking in methanol (1.5 L) for 48 h and filtering. The two filtrates were mixed and concentrated under reduced pressure at 40 °C.

The leaf extract was dissolved in methanol, and the dissolved extract was added to filter paper in a Petri dishes (2.8 cm i.d.). After the methanol in the Petri dishes was completely evaporated in a fume hood, 0.6 mL solution of Tween 20 (0.05% *w*/*v*, Nacalai, Kyoto, Japan) was applied to the filter paper. The seeds of *E. crus-galli*, *L. multiflorum* and *V. myuros* were germinated on another filter paper moistened with distilled water in the dark at 25 °C for 48 h. Ten germinated seeds of each species were then arranged on the filter paper in the Petri dishes. The length of the roots and coleoptiles of *E. crus-galli*, *L. multiflorum* and *V. myuros* was determined after the incubation of 48 h in the dark at 25 °C. The percentage length of the roots and coleoptiles of these weed species was calculated against the length of the roots and coleoptiles of the control plants. The control plants were treated using same procedure, with the only difference being not adding the leaf extracts to the filter paper in the Petri dishes. The allelopathic activity of the extracts was also determined with cress bioassay. The procedure of the cress bioassay was the same as for the weed species, but the seeds (not germinated) were arranged in the filter paper on the Petri dishes.

### 4.3. Separation of the F. suspensa Extract

The leaf extract of *F. suspensa* obtained as described in the above section was adjusted to pH 7.0 with a buffer of 1 M phosphate, and partitioned with ethyl acetate to separate the ethyl acetate and aqueous fractions (Figure 5). The ethyl acetate fraction was then applied to silica gel chromatography (silica gel 60, 70–230 mesh; Merck, Darmstadt, Germany). The allelopathic activity of all the separated fractions by the silica gel chromatography was determined using a cress bioassay as described above. The most active fraction 5 (Figure 6) was further subjected to a Sephadex LH-20 chromatography (Sigma-Aldrich, Burlington, VT, USA) and an ODS cartridge (YMC-Dispo SPE; YMC Ltd., Kyoto, Japan). Active compounds 1 and 2 were then isolated using a reverse-phase HPLC (Figure 7). The chemical structures of the compounds were determined by the spectrum analyses of HRESI-MS (Orbitrap Exploris 240, Thermo-Fisher K.K., Tokyo, Japan) and ^1^H-NMR (500 MHz, CDCl_3_; Bruker Avance III 500, Bruker Co., Yokohama, Japan), and optical rotation (P-1010 polarimeter, Jasco Co., Tokyo, Japan). All other reagents were purchased from Nacalai at a purity > 99.8.

### 4.4. Allelopathic Activity of the Isolated Compounds and Pinoresinol

(-)-Matairesinol and (-)-arctigenin were isolated from the *F. suspensa* leaves as described above. (+)-Pinoresinol was isolated from the leaves of *Osmanthus* × *fortunei* Carrière as described by [35]. A given quantity of the methanol solution of each compound was applied onto a filter paper in the Petri dish. After complete evaporation of the methanol solvent in the Petri dishes, 0.6 mL of a Tween 20 solution was added onto the filter paper. Then, 10 cress seeds and 10 germinated *L. multiflorum* seeds, were separately put on the filter paper and grown, and the allelopathic activity of the compounds was determined as described in the Section 4.2. The concentrations of the applied compounds were 0.1, 0.3, 1, 3 and 10 mM. 

### 4.5. Statistical Analysis

The bioassays were conducted with four replications using a completely randomized design with 10 plants for each determination. Significant differences between control and treatment were analyzed using Welch’s *t*-test (Figure 3, Figure 4 and Figure 6). The bioassay data for (-)-matairesinol, (-)-arctigenin and (+)-pinoresinol were subjected to a one-way analysis of ANOVA (SPSS, version 16.0, SPSS Inc., Chicago, IL, USA) and subsequent post-hoc analysis using Tukey’s HSD test at the *p* < 0.05 level (Figure 9, Figure 10 and Figure 11, Table 1). The *IC*_50_ values were obtained using GraphPad Prism 6.0 (Software of GraphPad, Inc., La Jolla, CA, USA).

## 5. Conclusions

The extracts of the *F. suspensa* leaves were allelopathic and significantly inhibited the growth of three weed species; *E. crus-galli*, *L. multiflorum* and *V. myuros* in vitro. (-)-Matairesinol and (-)-arctigenin were isolated and identified from the extracts as allelopathic agents. Both compounds inhibited the growth of *L. multiflorum* and cress in vitro, and the activity of (-)-arctigenin was greater than that of (-)-matairesinol. Moreover, (-)-arctigenin and (-)-matairesinol were more active than (+)-pinolesinol, which is their precursor in the biosynthetic pathway. The leaves may contain a large amount of (-)-matairesinol and (-)-arctigenin. When the leaves of *F. suspensa* were incorporated into the soil as a soil mixture, certain allelochemicals in the leaves may be liberated into the field soil during the decomposition process. The liberated allelochemicals may suppress the emergence and growth of some weed species. The leaf extracts and its soaking water can be applied as a foliar spray to inhibit the emergence and growth of some weed plant species. Therefore, because of its allelopathic activity, the leaves and extracts of *F. suspensa* are potentially useful for weed manegement options to reduce the application of synthetic herbicides in crop production and to develop to eco-friendly agriculture. However, evaluating the inhibitory activity of the leaf extracts of *F. suspensa* as a foliar spray, and the leaves in a soil mixture is necessary under field conditions.

## Figures and Tables

**Table 1:** *IC*_50_ values (mM) of (-)-matairesinol, (-)-arctigenin and (+)-pinoresinol against the growth of the roots and hypocotyls/coleoptiles of cress and *L. multiflorum.* Different letters on in the columns (same organ) indicate significant differences at the *p* < 0.05 level.

	Cress		*L. multiflorum*	
Compound	Root	Hypocotyl	Root	Coleoptile
(-)-Matairesinol	1.1 b	2.1 b	0.93 b	2.2 b
(-)-Arctigenin	0.79 a	1.3 a	0.85 a	1.3 a
(+)-Pinoresinol	2.1 c	2.3 b	1.1 c	2.5 b

## Data Availability

No supporting data in this study.
